# Pretransplant Physical Activity and Cardiovascular Risk Factors in Kidney Transplant Candidates: A Cross-Sectional Study

**DOI:** 10.3390/healthcare13101200

**Published:** 2025-05-20

**Authors:** Emilia Ferrer-López, Víctor Cantín-Lahoz, Francisco Javier Rubio-Castañeda, Juan José Aguilón-Leiva, María García-Magán, Carlos Navas-Ferrer, Eva Benito-Ruiz, María Isabel Serrano-Vicente, Isabel Blázquez-Ornat, Isabel Antón-Solanas, Fernando Urcola-Pardo

**Affiliations:** 1 Department of Physiatry and Nursing, Faculty of Health Sciences, University of Zaragoza, C/Domingo Miral s/n, 50009 Zaragoza, Spain; eferrerl@unizar.es (E.F.-L.); jaguilon@unizar.es (J.J.A.-L.); mgmagan@unizar.es (M.G.-M.); cnavasf@unizar.es (C.N.-F.); evabenitor@unizar.es (E.B.-R.); mariaisabel.serrano@unizar.es (M.I.S.-V.); iblazquez@unizar.es (I.B.-O.); furcola@unizar.es (F.U.-P.); 2 SAPIENF Research Group (B53-23R), Universidad de Zaragoza, C/Pedro Cerbuna, 12, 50009 Zaragoza, Spain; 3 Haemodialysis and Renal Transplant Unit, Hospital Universitario Miguel Servet de Zaragoza, Paseo Isabel la Católica 1-3, 50009 Zaragoza, Spain; victorcantin@gmail.com (V.C.-L.); fjrubio.due@gmail.com (F.J.R.-C.); 4 Instituto de Investigación Sanitaria de Aragón (IISA), Centro de Investigación Biosanitaria de Aragón (CIBA), C/San Juan Bosco, 13, 50009 Zaragoza, Spain

**Keywords:** chronic kidney disease, kidney transplantation, physical activity, preoperative exercise, waiting list

## Abstract

**Background/Objectives:** Individuals with chronic kidney disease often face significant physical and clinical challenges, such as muscle weakness, fatigue, and reduced cardiorespiratory capacity, that impact their quality of life. Physical activity has emerged as an effective intervention to counteract these effects, with clinical guidelines recommending exercise as a standard treatment for kidney transplant recipients. The aim of this study was to assess pretransplant physical activity levels in a cohort of transplant patients and analyze their relationships with cardiovascular risk factors. **Methods:** A cross-sectional, analytical, and correlational study was conducted from September 2020 to June 2022 with a sample of 122 kidney transplant recipients assessed before kidney transplantation. Sociodemographic data, anthropometric data, comorbidities, renal replacement therapy types, and clinical and analytical data were collected from the patients’ clinical records. Physical activity was assessed via the International Physical Activity Questionnaire. **Results:** The average time spent waiting for transplantation was 423 ± 405 days, which was longer (387 ± 524) in the group of those under 65 years than in those over 65 years (194 ± 256) (*p* = 0.010). The median energy expenditure was 1742 (IQR = 1719) METs. In addition, 15.6% of the participants reported inactivity. Men reported higher physical activity levels (median: 2076 METs/week; IQR: 2037) than women did (median: 1386 METs/week; IQR: 1238). A higher level of physical activity was found in non-dialysis patients, overweight patients, and those with a history of stroke. A significant positive correlation was found between physical activity levels and serum urea. **Conclusions:** Increased physical activity levels were observed in men and in participants under 65 years of age. Patients with cardiovascular risk factors, such as hypertension, diabetes mellitus, dyslipidemia, overweight and obesity, reported lower activity levels, whereas those with a prior history of cerebrovascular accidents engaged in more physical activity. This study highlights the importance of assessing physical activity and promoting exercise for chronic kidney disease patients awaiting kidney transplantation. Further research is needed to explore the evolution of physical activity in this population and its impact post-transplantation.

## 1. Introduction

Kidney transplantation is the best therapeutic option for select patients with chronic kidney disease (CKD). Patients with CKD experience a number of physical and metabolic alterations that impact their quality of life and their ability to perform daily activities [1,2,3,4]. In this context, physical activity (PA) has emerged as an effective nonpharmacological intervention to mitigate some of the negative effects of the disease. Most clinical practice guidelines (CPGs) recommend structured exercise programs as standard care for organ transplant recipients. Exercise helps minimize the effects of muscle weakness and fatigue, which are common in patients with advanced kidney disease [5]. In addition, promoting an active lifestyle in this population is essential to reduce sedentary behavior, improve cardiovascular function, and enhance overall quality of life [6,7].

Among other modifiable lifestyle risk factors, physical inactivity is suspected to play a major role in most chronic diseases. Specifically, it is associated with worse renal function and decreased survival posttransplant [8,9,10], as inactive patients present lower muscle mass and worse outcomes posttransplant [11,12]. Conversely, transplant recipients who engage in regular PA have better outcomes, including higher aerobic capacity, muscle strength, and quality of life [13,14,15].

Currently, kidney transplant candidates tend to be older and often present a higher prevalence of comorbidities, such as hypertension, dyslipidemia, obesity and diabetes, along with modifiable risk factors such as physical inactivity, all of which substantially increase the risk of cardiovascular disease (CVD) [8,16]. In this context, physical exercise in patients with CKD, particularly those on the waiting list for kidney transplantation, has generated increasing research interest in recent years. This is due to the potential benefits it offers in improving emotional well-being and quality of life, contributing to the management of stress, anxiety and depression linked to the waiting process [17,18].

Recent studies have increasingly focused on the role of PA in patients awaiting kidney transplantation [19]. Evidence suggests that higher pretransplant PA levels are associated with better posttransplant outcomes, such as improved functional capacity, reduced hospitalization time, and enhanced graft survival [20]. However, despite these benefits, PA levels among transplant candidates often remain low, highlighting the need for prehabilitation interventions specifically targeting this population [21].

### Background

Traditionally, physical exercise has not been recommended for patients with CKD because of the possibility of further impairing renal function and increasing proteinuria [22]. Today, sedentary lifestyles can be both a cause and a consequence of kidney disease progression, and physical exercise decreases as the glomerular filtration rate worsens [23]. Despite the limitations that CKD may impose, studies have shown that moderate, adapted PA can significantly improve patients’ physical fitness; reduce the risk of cardiovascular and metabolic complications, osteoporosis and sarcopenia, among other conditions; and promote a better response to transplantation [24,25]. PA has become a mainstay of public health strategies and programs in recent decades because of its numerous benefits and the recognition that physical inactivity is the fourth leading risk factor for global mortality, as supported by recent research [26,27,28] and official reports from the World Health Organization [29]. Nevertheless, the first comprehensive exercise guidelines specifically designed for CKD populations—including patients on conservative treatment, dialysis, and kidney transplant recipients (RTRs)—were developed in 2020 by the UK Renal Research Consortium Clinical Study Group for Exercise and Lifestyle. These guidelines recommend the integration of tailored exercise programs adapted to each clinical situation to enhance functional capacity, cardiovascular health, and transplant outcomes. Recent investigations [30,31] have suggested that sufficient PA before and after transplantation can reduce all-cause and cardiovascular mortality and that exercise interventions before surgery (prehabilitation) can help increase pretransplant PA levels and aid in posttransplant recovery.

Additionally, the KDIGO Guidelines for the Evaluation and Management of CKD recommend that patients with good cardiovascular health engage in at least 30 min of moderate activity five times per week (equivalent to 450–750 MET-min/week) as part of a broader strategy to maintain a healthy BMI (between 20 and 25 kg/m^2^), promote a healthy lifestyle, and cease smoking to reduce cardiovascular risk [1]. The World Health Organization (WHO) defines PA as any movement of the body produced by the contraction of skeletal muscles that increases energy expenditure compared with the baseline value (e.g., walking, climbing stairs) [32]. Assessing the type of PA performed by CKD patients and reducing sedentary behavior are essential steps in determining their baseline activity levels before introducing safe and tailored exercise programs [23].

However, despite growing interest in the benefits of exercise post transplantation, evidence regarding the role of PA in the pretransplant phase remains limited [33]. Few studies have specifically evaluated how pretransplant PA levels influence patient outcomes, including postoperative recovery, graft survival, and long-term cardiovascular risk [34]. Furthermore, interventions targeting PA during the waiting period are still scarce, representing an important gap in current clinical practice and research [35,36]. Therefore, a deeper understanding of pretransplant PA patterns is essential to guide future strategies aimed at optimizing transplant outcomes from the earliest stages of patient care [35].

As we hypothesized that PA levels among kidney transplant candidates would vary according to demographic and clinical factors—with lower activity levels expected among patients with cardiovascular risk factors and higher levels among younger and male participants—this study aimed to assess pretransplant PA levels in a prospective cohort of transplant patients and analyze their relationship with cardiovascular risk factors such as hypertension, diabetes mellitus, cardiovascular events, dyslipidemia, overweight, and obesity.

## 2. Materials and Methods

### 2.1. Study Design and Participants

A cross-sectional, analytical, and correlational study was carried out at the transplant service of the Hospital Miguel Servet in Zaragoza (Spain). The study population comprised every kidney transplant recipient who underwent grafting at said hospital from September 2020 to June 2022. All the data were collected during the preoperative hospitalization phase, prior to transplantation. This study followed the STROBE’s recommendations for reporting observational studies [37].

**Figure 1:**
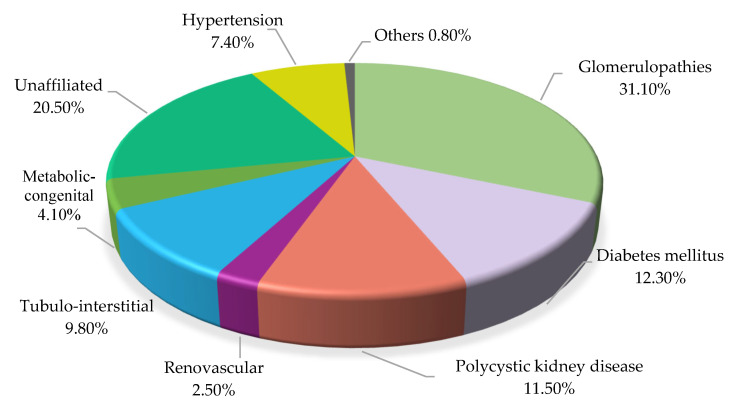
Pathologies leading to kidney failure.

The inclusion criteria required participants to be 18 years or older on the date of surgery and to have signed the informed consent form. The exclusion criteria encompassed any conditions that hindered effective communication, such as hearing or speech impairments, and difficulties with reading or writing. All patients included in the study had a diagnosis of CKD as the underlying condition. However, while specific etiological diagnoses leading to kidney failure were recorded (Figure 1), they were not considered independent variables, as this was not an objective of the study.

### 2.2. Variables and Data Collection

Sociodemographic and clinical variables, including age, sex, anthropometric measures (height, weight, body mass index (BMI), obesity rate), relevant comorbidities (hypertension, diabetes, dyslipidemia, cardiovascular disease, cardiorespiratory disease, cerebrovascular accident), toxic habits (tobacco, ethanol consumption), waiting time until transplantation, type of pretransplant renal replacement therapy (no dialysis, peritoneal dialysis, hemodialysis), and analytical values (urea, creatinine, total protein, albumin, ferritin, hemoglobin, hematocrit, HbA1c), were obtained from the participants’ clinical records.

PA was assessed via the International Physical Activity Questionnaire (IPAQ) [38]. Patients aged 18–65 years answered the standard Spanish version [39], whereas patients over 65 years completed the elderly version (IPAQ-E) [40]. The questionnaire captured the PA level before transplantation through personal interviews by the principal investigator before hospital discharge. Metabolic equivalent units (METs) were calculated to estimate energy expenditure per minute per week (MET-min/week).

METs are multiples of the basal metabolic rate and are defined as the minimum energy expenditure required to maintain vital physiological functions at rest [41]. Due to the nonnormal data distribution, medians and interquartile ranges were used for energy expenditure analysis. PAs were categorized into low (category 1), moderate (category 2), and high (category 3) (Table 1).

### 2.3. Data Analysis

For the descriptive analysis, the results of the qualitative variables are presented in terms of absolute and relative frequency distributions (n, %), whereas the quantitative variables are presented via measures of central tendency and dispersion (means and standard deviations for those following a normal distribution and medians and interquartile ranges (IQRs) for those with a nonnormal distribution). Inferential analysis was conducted by comparing proportions when both variables were qualitative (using the chi-square test and Fisher’s exact test) and by comparing means or ranks for quantitative variables. Parametric tests (Student’s *t* test and ANOVA) were applied for normally distributed data, and nonparametric tests (Mann-Whitney U test or Kruskal-Wallis test) were used for nonnormally distributed data.

Given the positive skewness observed in the distribution of MET-min/week values derived from the IPAQ, an inverse transformation of the dependent variable was applied prior to conducting exploratory multivariate regression analysis to meet the assumptions required for linear regression models. The need for the transformation of physical activity measures obtained through the IPAQ to address nonnormality has been documented in previous research [42,43], and the application of inverse regression methods in skewed datasets has been validated in the statistical literature [44].

Differences were considered significant at *p* < 0.05 with a 95% confidence interval (CI). The statistical analysis was performed via SPSS v.27.

### 2.4. Ethical Issues

Before data collection, the participants were informed about the investigation’s aims and procedures. They voluntarily agreed to participate and provided written consent. The participants were also informed that they could withdraw from the study at any time without consequences. Permission for the use of the IPAQ and the IPAQ-E questionnaires was obtained from the respective authors. The researchers adhered to the Declaration of Helsinki (WHO, 2013), ensuring anonymity by omitting patient names, initials, and medical records. This study was authorized by the hospital’s management and approved by the Research Ethics Committee of Aragón (C.P.-C.I. PI20/278).

## 3. Results

### 3.1. Descriptive Analysis

A total of 122 kidney transplant recipients were included in the study, with data collected during their preoperative hospitalization phase. More than two-thirds (70.5%) were men, with an average age of 56.66 years (SD = 14.51), ranging from 18 to 80 years; most were younger than 65 years old (68%). Among these patients, 67.2% received renal replacement therapy (RRT) through hemodialysis (HD), 25.4% through peritoneal dialysis (PD), and 7.4% were in a non-dialysis state. Almost half had an arteriovenous fistula for vascular access (45%), and most were undergoing their first kidney transplant (85.2%).

Regarding clinical comorbidities, most patients had arterial hypertension (91.8%), while half had dyslipidemia (50.8%) and/or a BMI over 25 kg/m^2^, with 34.4% being overweight and 16.4% being obese. More than a quarter had ischemic heart disease (27.0%), almost a fifth had diabetes (19.7%) or a toxic habit (18.2%), 14.8% had respiratory disease, and 4.9% had a history of cerebrovascular accident.

The average time spent on the waiting list for transplantation was 423 ± 405 days (min: 1, max: 2023). Women spent less time on the waiting list, with no statistically significant differences found. Statistically significant differences were found (*p* = 0.010) when comparing time spent on the waiting list by individuals under 65 (387 ± 524 days) and those aged 65 or over (194 ± 256 days; min: 15, max: 2279). A statistically significant difference in weight was observed between men and women, with a greater proportion of men being overweight (*p* < 0.05) (Table 2).

The analytical values revealed above-normal means for ferritin (384.85 ng/mL; SD = 458.62), creatinine (6.08 mg/dL; SD = 3.03), and serum urea (106.50 mg/dL; SD = 80.0). Normal values were observed for total protein (6.65 g/dL; SD = 0.83), albumin (4.00 g/dL; SD = 0.52), and HbA1c (5.30%; SD = 0.70), whereas slightly low levels were found for hemoglobin (12.17 g/dL; SD = 1.38) and hematocrit (35.80%; SD = 4.92). A significant sex-based difference was found in the serum creatinine levels, with women presenting lower values than men did (*p* = 0.01) (Table 3).

The analytical variables showed that 92% of patients had urea levels above the reference values. All patients (100%) had elevated creatinine levels. For ferritin, 2% and 54% of patients had concentrations below and above the recommended values, respectively, whereas 9% had elevated glycated hemoglobin. In addition, 16% of patients had albumin deficiency, with 50% showing total protein levels below the recommended values and 49% being within the normal range. In terms of sex, a greater percentage of women (86.1%) than men (81.4%) had hemoglobin levels below the recommended values. Eighty-six percent of women and 85% of men had hematocrit values below the recommended values.

Regarding PA, the patients in our sample reported a median of 1742 MET-min/week (IQR = 1719). According to the IPAQ classification, more than half of the participants (53.3%) were classified as engaging in high levels of PA, with a median of 2772 MET-min/week (IQR = 2240). Additionally, 31.1% of the participants were classified as having a moderate level of PA, with a median of 1386 MET-min/week (IQR = 346.13). Finally, 15.6% were classified as having a low level of PA, with a median of 393 MET-min/week (IQR = 306) (Table 4).

### 3.2. Inferential Analysis

When examining PA in relation to qualitative independent variables, notably higher levels of PA were observed among male participants, those aged below 65 years, non-dialysis patients, those with vascular access through an arteriovenous fistula, and those with excess body weight or a history of prior transplantation. Higher PA levels were also noted among normotensive subjects, individuals without a history of diabetes or dyslipidemia, and those without ischemic heart disease or respiratory ailments.

Similarly, patients who exhibited toxic habits and those with a history of cerebrovascular disorders presented increased PA levels (2079 (IQR = 2274) and 3462 (IQR = 3533) MET-min/week, respectively). Notably, the sole statistically significant disparity emerged within the latter group (*p* = 0.035). Nevertheless, it is prudent to exercise caution when interpreting this finding because of disparities in group sizes (Table 5).

When the correlations between the participants’ PA and the quantitative independent variables were examined, a statistically significant positive correlation was observed between the MET values and the participants’ serum urea levels (r = 0.204; *p* < 0.05). The remaining variables analyzed did not show significant correlations with the PA values (Table 6).

Finally, a new MET variable was created by inverse transformation to perform an explorative multivariate regression analysis, with the independent variables sex, age, time on the waiting list and BMI; however, no statistically significant differences were found by sex (*p* = 0.057), age (*p* = 0.491), time on the waiting list (*p* = 0.579) or BMI (*p* = 0.679).

## 4. Discussion

The sociodemographic characteristics and cardiovascular risk profiles of our participants are consistent with those reported in previous studies on kidney transplant candidates, specifically reflecting a predominance of male patients with chronic kidney disease [45,46,47]. In addition, a higher incidence of end-stage renal disease was also observed in men, with women experiencing a slower progression of CKD [48,49]. Several studies have reported sex disparities in the treatment of chronic kidney disease (CKD) and renal replacement therapy (RRT). Interestingly, women are more likely to donate a kidney during their lifetime [50], yet they are less likely to receive a transplant or undergo dialysis than men are [49,51,52]. The present study revealed a shorter waiting time for women than for men, which contrasts with previous research suggesting that women typically experience longer waiting periods for transplantation than men do [53].

In our study, more than 30% of the patients were over 65 years of age. This proportion is higher than that reported in other studies, in which between 15% and 20% of renal transplants were performed in patients over 65 years. This pattern was also observed in other studies, with figures varying depending on the region and the selection criteria [54,55,56,57,58,59]. Notably, more than half of our sample reported engaging in vigorous PA, which contrasts with other studies in which pretransplant PA levels were less than 10%; additionally, moderate PA was high compared with that reported in Rosas’ study (14–18%) [60].

The inactivity rates observed in our sample were also lower than those reported in previous studies, where higher levels of inactivity were noted [21,61]. These differences could be partially explained by the variability in physical activity assessment instruments, as the IPAQ captures activity bouts of at least 10 min, potentially leading to higher estimated MET-min/week values [10]. However, it is also important to consider that public health strategies and clinical practice guidelines promoting physical activity in patients with chronic kidney disease have evolved significantly in recent years. Most of the data from the studies used for comparison were collected before 2015 (Rosas et al., 2000–2004 [60]; Masiero et al., 2002–2015 [21]; Wilkinson et al., 2012–2018 [61]), whereas the last decade has seen stronger recommendations encouraging regular exercise for CKD patients [62], particularly given that a lower pretransplant physical functioning score is significantly associated with a higher risk of hospitalization and death posttransplant [63]. In addition, cultural and regional factors promoting active lifestyles, as well as possible educational programs aimed at transplant candidates in Spain, could have contributed to the higher reported levels of physical activity observed in our cohort [64,65].

Our findings align with those of prior studies in which women and individuals over 65 years of age presented lower levels of PA than did sociodemographic variables [21,24]. Notably, PA increased significantly among patients with a history of stroke, supporting recommendations for therapeutic exercise poststroke [66]; however, in contrast, Kaysen reported lower physical performance in patients with this condition [67].

Although the observed positive correlation between physical activity and serum urea levels might be contradictory, it may be explained by higher muscle mass and better nutritional status among more active patients. Increased muscle metabolism and protein turnover associated with greater physical activity could contribute to higher serum urea concentrations without necessarily indicating impaired renal function. This phenomenon has been observed both in human exercise physiology and in studies assessing performance in endurance horses, where elevated urea reflects increased nitrogen metabolism due to increased muscle activity rather than renal deterioration [3,68]. In patients with chronic kidney disease (CKD), serum urea levels should therefore be interpreted cautiously, as they may reflect functional status, muscle mass, and physical exertion rather than disease severity alone [69,70,71]. Contextual factors such as hydration status and overall protein metabolism are essential considerations when evaluating these biomarkers [72].

There have been numerous noncontrolled and controlled studies of aerobic and/or resistance exercise programs in patients with CKD (>40 studies) and in those receiving HD (approximately 1000 patients), but very few have focused on patients actually listed for transplantation [73]. Candidates often face long waits, leading to declines in functional capacity due to aging, chronic conditions, frailty risk, and dialysis stress [55,56]. Regular low-to-moderate PA during hemodialysis also benefits patients without adverse effects, preventing overweight and obesity and reducing stress, which is beneficial for kidney transplant recipients [74,75,76,77,78].

Although no statistically significant differences were found, our results suggest a negative correlation between waiting time on the transplant list and physical activity levels. This finding is consistent with those of previous studies, such as those by Kang [24] and Pinillos-Patiño [79], which also reported a progressive decrease in physical activity associated with longer disease duration, whether measured as the time since CKD diagnosis or the time of dialysis. These results collectively support the notion that prolonged disease progression negatively impacts physical activity among kidney transplant candidates. However, transplant candidates may be motivated to exercise in anticipation of surgery, with pretransplant interventions improving physical function, weight control, and attitudes toward PA [18,80,81].

Nurses are a fundamental pillar in promoting PA through education [81], not only in terms of its implementation but also in terms of tailoring their recommendations on the basis of their patients’ preferences through the exploration of facilitators and barrier factors [82]. As our results support early assessment and promotion of PA among transplant candidates, person-centered care recommendations should improve adherence to PA, with a clear focus on well-being and quality of life after transplantation [83].

### Limitations

We identified several limitations. First, no information was collected on patients’ quality of life before transplantation. Additionally, the growing promotion of PA and the increasing availability of information about its benefits may cause the population to overestimate their perceived energy expenditure. Another limitation is the use of self-report questionnaires to assess physical activity. Although validated and widely used, questionnaires such as the IPAQ may introduce recall bias and subjective inaccuracies compared with objective measurements such as those obtained via accelerometers. Nevertheless, the use of accelerometers also presents limitations, including economic constraints and the challenges associated with device placement and compliance among patients awaiting transplantation. Finally, our study did not assess dietary habits or adherence to nutritional recommendations. Although nutritional counseling is an essential component of renal replacement therapy, each modality involves specific dietary guidelines, and evaluating their implementation was beyond the scope of this study.

## 5. Conclusions

Our findings suggest that men and individuals younger than 65 years perform high levels of PA. Participants with cardiovascular risk factors such as hypertension, diabetes, dyslipidemia, and respiratory and cardiovascular diseases reported lower levels of PA, whereas patients without dialysis, individuals with a previous transplant, overweight individuals and those with a prior history of cerebrovascular accidents presented higher levels of PA.

Recognizing the clinical impact of low PA levels is essential for developing and implementing more effective strategies to assess physical fitness and promote early exercise interventions in this population. Our findings highlight the importance of initiating personalized PA recommendations from the moment of inclusion on the transplant waiting list, with a particular focus on vulnerable groups exhibiting lower levels of PA. Furthermore, longitudinal studies that examine the evolution of PA patterns in CKD patients and evaluate the long-term impact of kidney transplantation on PA behaviors and clinical outcomes are needed.

## Figures and Tables

**Table 1:** PA categories according to the IPAQ [38].

Category	Characteristics
Low Category 1 (<600 METs)	No PA. Insufficient PA to reach categories 2 (Moderate) or 3 (High).
Moderate Category 2 (600–1499 METs)	Moderate PA and/or walking on five or more days for at least 30 min each day OR Combination of walking and/or moderate to vigorous PA, achieving an energy expenditure of at least 600 METs per minute and per week on five or more days OR Vigorous PA on three or more days for at least 25 min each day.
High Category 3 (>1500 METs)	Combination of walking and/or moderate to vigorous PA on seven or more days per week, achieving an energy expenditure of at least 3000 METs per minute and per week ORVigorous PA on at least three days per week, achieving an energy expenditure of 1500 METs per minute and per week.

**Table 2:** Baseline sociodemographic and clinical characteristics.

Variable	Category	n (%)	Men	Women	*p*
		122 (100%)	86 (70.5%)	36 (29.5%)	
Age (categorized)	18–64	83 (68.0%)	59 (48.4%)	24 (19.7%)	0.682
	≥65	39 (32.0%)	27 (22.1%)	12 (9.8%)	0.221
Renal replacement therapy	Nondialysis	9 (7.4%)	6 (4.9%)	3 (2.5%)	0.622
	Peritoneal dialysis	31 (25.4%)	24 (19.7%)	7 (5.7%)	
	Hemodialysis	82 (67.2%)	56 (45.9%)	26 (21.3%)	
Vascular access	No vascular access	40 (32.8%)	30 (24.6%)	10 (8.2%)	0.205 ^+^
	Arteriovenous fistula	55 (45.1%)	40 (32.8%)	15 (12.3%)	
	Catheter	27 (22.1%)	16 (13.1%)	11 (9.0%)	
Previous transplant	Non-previous transplant	104 (85.2%)	74 (60.7%)	30 (24.6%)	0.781 ^+^
	Previous transplant	18 (14.8%)	12 (9.8%)	6 (4.9%)	
BMI	Underweight	6 (4.9%)	2 (1.6%)	4 (3.3%)	0.013 ^+^
	Normal weight	54 (44.3%)	34 (27.9%)	20 (16.4%)	
	Overweight	42 (34.4%)	36 (29.5%)	6 (4.9%)	
	Obesity	20 (16.4%)	14 (11.5%)	6 (4.9%)	
Patients with hypertension		112 (91.8%)	86 (64.8%)	36 (27.0%)	0.972
Patients with previous diabetes		24 (19.7%)	17 (13.9%)	7 (5.7%)	0.967
Patients with dyslipidemia		62 (50.8%)	44 (71.0%)	18 (29%)	0.907
Patients with ischemic heart disease		33 (27.0%)	23 (18.9%)	36 (8.2%)	0.907
Patients with respiratory disease		18 (14.8%)	16 (13.1%)	2 (1.6%)	0.064
Patients with cerebrovascular accident		6 (4.9%)	4 (3.3%)	2 (1.6%)	0.574 ^+^
Patients with toxic habits		22 (18.2%)	16 (13.2%)	6 (5.0%)	0.850
		**Median (IRQ)**	**Men**	**Women**	
Waiting list time in days		423 (405)	381 (471)	253 (499)	0.227

^+^ Fisher’s exact test.

**Table 3:** Blood and urine parameters.

	Mean (SD)	Men	Women	*p*
Ferritin (15–200 ng/mL)	384.85 (458.62)	384.85 (471.42)	384.85 (417.12)	0.650
Creatinine (0.51–0.95 mg/dL)	6.08 (3.03)	6.27 (3.90)	5.33 (2.95)	0.010
Urea (17–43 mg/dL)	106.50 (80.0)	110 (81.50)	102 (75.75)	0.148
Protein (6.6–8.3 g/dL)	6.65 (0.83)	6.7 (0.90)	6.6 (0.80)	0.331
Albumin (3.5–5.2 g/dL)	4 (0.52)	4 (0.60)	3.90 (0.58)	0.168
Hemoglobin (g/dL)	12.17 (1.38)	12.10 (1.82)	11.70 (2.10)	0.415
Hematocrit (%)	35.80 (4.92)	36 (4.80)	35.60 (18.20)	0.678
Glycated hemoglobin (HbA1c)	5.30 (0.70)	5.30 (0.70)	5.30 (0.78)	0.280

**Table 4:** PA values (IPAQ classification).

	Total	High PA	Moderate PA	Low PA
n (%)	122 (100%)	65 (53.3%)	38 (31.1%)	19 (15.6%)
MET-min/week	1742 (1719)	2772 (2240)	1386 (346.13)	393 (306)

**Table 5:** PA analysis according to qualitative variables.

Variable	Category	n (%)	Median (IQR)	*p*
Sex	Men	86 (70.5%)	2076 (2037)	0.062 ^U^
	Women	36 (29.5%)	1386 (1238)	
Age	18–64	83 (68%)	1746 (1557)	0.925 ^U^
	≥65	39 (32%)	1533 (2340)	
Renal replacement therapy	No dialysis	9 (7.4%)	2619 (1386)	0.574 ^+^
	Peritoneal dialysis	31 (25.4%)	1626 (1601)	
	Hemodialysis	82 (67.2%)	1739 (1922)	
Vascular access	No vascular access	40 (32.8%)	1741 (1575)	0.912 ^+^
	Arteriovenous fistula	55 (45.1%)	1848 (1879)	
	Catheter	27 (22.1%)	1466 (2538)	
BMI	Underweight	6 (4.9%)	1213 (1869)	0.232 ^+^
	Normal weight	54 (44.3%)	1562 (1732)	
	Overweight	42 (34.4%)	2016 (2666)	
	Obesity (BMI ≥ 30)	20 (16.4%)	1426 (1439)	
Previous transplant	Yes	104 (85.2%)	1913 (2027)	0.483 ^U^
	No	18 (14.8%)	1735 (1646)	
Hypertension	No	10 (8.2%)	2086 (3878)	0.940 ^U^
	Yes	112 (91.8%)	1742 (1559)	
Previous diabetes	No	98 (80.3%)	1762 (1719)	0.799 ^U^
	Yes	24 (19.7%)	1506 (1520)	
Dyslipidemia	No	60 (49.2%)	1764 (1784)	0.501 ^U^
	Yes	62 (50.8%)	1682 (1704)	
Ischemic heart disease	No	89 (73%)	1782 (1550)	0.611 ^U^
	Yes	33 (27%)	1386 (2754)	
Respiratory disease	No	104 (85.2%)	1746 (1796)	0.831 ^U^
	Yes	18 (14.8%)	1426 (1213)	
Cerebrovascular accident	No	116 (95.1%)	1633 (1646)	0.035 ^U^
	Yes	6 (4.9%)	3462 (3533)	
Toxic habits	No	100 (81.8%)	1737 (1606)	0.903 ^U^
	Yes	22 (18.2%)	2079 (2274)	

^U^ Mann–Whitney; ^+^ Kruskal–Wallis.

**Table 6:** Pearson correlations between PA and quantitative variables.

Variable	Correlation Value	*p*
Age	0.008	0.927
BMI	0.122	0.182
Waiting list time	−0.018	0.846
Ferritin	−0.020	0.824
Creatinine	0.081	0.376
Urea	0.204	0.024
Protein	0.106	0.246
Albumin	0.009	0.921
Hemoglobin	0.081	0.377
Hematocrit	0.101	0.267
Glycated hemoglobin	0.099	0.280

## Data Availability

The data presented in this study are available upon request to the corresponding author due to confidentiality reasons.

## Abbreviations

The following abbreviations are used in this manuscript:

BMI	Body Mass Index
CI	Confidence Interval
CKD	Chronic Kidney Disease
CPG	Clinical Practice Guideline
CVD	Cardiovascular Disease
HD	Hemodialysis
IPAQ	International PA Questionnaire
IQR	Interquartile Range
KDIGO	Kidney Disease Improving Global Outcomes
MET	Metabolic Equivalent Unit
PA	Physical Activity
PD	Peritoneal Dialysis
RRT	Renal Replacement Therapy
WHO	World Health Organization
